# Spectral properties of physiological mirror activity: an investigation of frequency features and common input between homologous muscles

**DOI:** 10.1038/s41598-022-20413-2

**Published:** 2022-09-24

**Authors:** Rouven Kenville, Tom Maudrich

**Affiliations:** 1 Department of Movement Neuroscience, Faculty of Sports Science, Leipzig University, 04109 Leipzig, Germany; 2 Department of Neurology, Max Planck Institute for Human Cognitive and Brain Sciences, 04103 Leipzig, Germany

**Keywords:** Motor control, Sensorimotor processing

## Abstract

During unilateral contractions, muscular activation can be detected in both active and resting limbs. In healthy populations, the latter is referred to as physiological mirror activity (pMA). The study of pMA holds implications for clinical applications as well as the understanding of bilateral motor control. However, the underlying mechanisms of pMA remain to be fully resolved. A commonality of prevailing explanatory approaches is the concept of shared neural input. With this study, we, therefore, aimed to investigate neural input in the form of multiple analyses of surface electromyography (sEMG) recordings in the frequency domain. For this purpose, 14 healthy, right-handed males aged 18–35 years were recruited. All participants performed a pinch-force task with the dominant hand in a blockwise manner. In total, 9 blocks of 5 contractions each were completed at 80% of maximum force output. Muscle activity was recorded via sEMG of the first dorsal interosseous muscle of the active and resting hand. We analyzed (1) spectral features as well as (2) intermuscular coherence (IMC). Our results demonstrate a blockwise increase in median frequency, mean frequency, and peak frequency in both hands. Frequency ratio analyses revealed a higher low-frequency component in the resting hand. Although we were able to demonstrate IMC on an individual level, results varied greatly and grand-averaged IMC failed to reach significance. Taken together, our findings imply an overlap of spectral properties between active and passive hands during repeated unilateral contractions. Combined with evidence from previous studies, this suggests a common neural origin between active and resting hands during unilateral contractions possibly resulting from a reduction in interhemispheric inhibition due to high force demands. Nevertheless, the exploratory nature of this study necessitates the classification of our results through follow-up studies.

## Introduction

Physiological mirror activity (pMA) describes unintentional muscle activity in resting limbs during unilateral contractions in neurologically healthy populations, that can be observed through surface electromyographic (sEMG) recordings. Inherent properties of pMA have been primarily studied by way of time-domain analyses of sEMG. To date, it is well known and often replicated, that pMA amplitude changes as a function of the amount of applied force during unilateral movements^1–8^. Factors influencing pMA amplitude apart from force requirements include increased movement frequency^9^ and rhythmicity^10^, increased cognitive load induced by simultaneously provided visual stimuli during task execution^11^, executive and attentional processes^12,13^, as well as central and peripheral fatigue resulting from repetitive exhaustive contractions^14–18^. Recently, it was further demonstrated that pMA is not time-locked to the onset of voluntarily contracting hand muscles but starts with varying and dynamically changing latencies, which are inversely correlated with respective amplitudes^18^.

Nevertheless, unresolved aspects of pMA remain, most prominently its neural origin^15^. Numerous explanatory approaches exist, for example, the theory of motor overflow^19,20^ or motor irradiation^17^, which assumes that pMA results from an ongoing modulation of interhemispheric communication during unilateral contractions with increasing force demands. These modulations lead to a shift from interhemispheric inhibition (IHI) to interhemispheric facilitation (IHF), which in turn induces bilateral activation of motor-relevant brain regions^4,19^. Alternative viewpoints suggest that ipsilateral projections of the corticospinal tract (CST) lead to bilateral activation in limbs both contralateral and ipsilateral to the cortical origin of the motor command^15^. A unifying feature of these theories is the question of the neural drive underlying active and resting limbs, i.e., the extent to which both effectors share neural input. In addition to time-domain analyses, frequency-domain analyses of sEMG allow insights into the neural drive to muscles by providing information on signal power distribution^21^, central and peripheral fatigue^22^, as well as fibre type characteristics^23^. Furthermore, quantifiable properties of sEMG frequency content, referred to as spectral features, have proven to be useful in the description and classification of physiological and pathophysiological muscle activities^24^. Among the commonly studied frequency features are median frequency (MDF), mean frequency (MNF), peak frequency (PKF), and frequency ratio (FR). MDF, MNF, and PKF are mainly used to assess fatigue-related effects^22^, whereas FR can be used to study the relative contribution of low and high frequency components to total signal power^25^. Each of these spectral features covers a particular aspect of the sEMG frequency content, thus aiding in the assessment of sEMG signals. Frequency analyses can be performed within a single signal, but can also be extended to multiple related signals. A well-established method in this context is intermuscular coherence (IMC). IMC enables the study of functional binding between muscles^26^. Such binding between muscles was first thought to reflect a common neural drive underlying functionally related muscle activities^27^. It was later demonstrated, that common oscillatory input to a set of motoneurons is present at the output of these motoneurons^21^ and more importantly emphasized, compared to other non-linear inputs, in the common neural drive to the muscle^28,29^. IMC can, therefore, be used to reveal the presence or absence of a common descending drive between functionally related muscles^26^.

In summary, a systematic analysis of (1) frequency content in active and resting hands during unilateral contractions, as well as (2) shared frequency content between active and resting hands has yet to be conducted. Such multimodal analyses in the frequency domain represent a promising extension to the study of pMA. Therefore, in this study, we aimed to investigate both the spectral features and IMC of active and resting limbs to contribute to the uncovering of their physiological origin. Here, we focussed on pMA of the upper extremities, particularly the first dorsal interosseous (FDI), as it is the best-studied muscle in relation to this phenomenon. Regarding spectral features, we hypothesized differences in terms of reduced MNF, MDF, and PKF as well as a higher proportion of low-frequency components in the resting compared to the active hand. This was mainly motivated by the fact that pMA has fundamentally lower amplitudes in the noncontracting muscle compared with the contracting muscle^4,5^. Furthermore, we hypothesized shared neural drive of both muscles and thus the presence of IMC based on contemporary theories on the origin of pMA. Perspectively, the results of this study apply to patients as well as healthy individuals, as the study of pMA has implications not only for clinical applications but also for understanding bimanual motor control in general.

## Materials and methods

This study partly represents a reanalysis of previously published sEMG data^18^.

### Ethical approval

The study was endorsed by the Ethics Committee at the Medical Faculty of Leipzig University (ref. no. 429/15-ff). All participants gave written informed permission to participate in the tests and were compensated for involvement. All methods were carried out in accordance with the Declaration of Helsinki.

### Participants

Twenty-four healthy male participants were enrolled in this study. Since a detailed description of frequency-related parameters requires the presence of pMA, only participants with robust, visible pMA across all trials were analyzed in this study. Specifically, only participants with observable pMA, i.e. EMG activity exceeding mean baseline activity + 2 standard deviations for a time window of at least 10 ms, over each of the 45 contractions for at least 1 s of the 3-s contraction durations were selected to ensure actual analysis of pMA. Accordingly, fourteen healthy male participants were included in this study (age: (mean ± standard deviation) 27.2 ± 4.6 years). Only male participants were recruited to account for possible gender-related differences in brain structure and function^30^, which may have influenced the motor behavior under investigation. All participants were right-handed according to the Oldfield handedness inventory^31^ (laterality quotient: 86.4 ± 18.9) and none of them had any history of playing musical instruments. None of the participants followed an organized training regime but performed general physical activities (3.9 ± 2.1 h per week). In addition, participants were advised to prevent alcohol and caffeine consumption 24 h before the experimental sessions due to its well-known influence on force production and the functioning of the central nervous system^32^.

### Behavioral experiment

**Figure 1:**
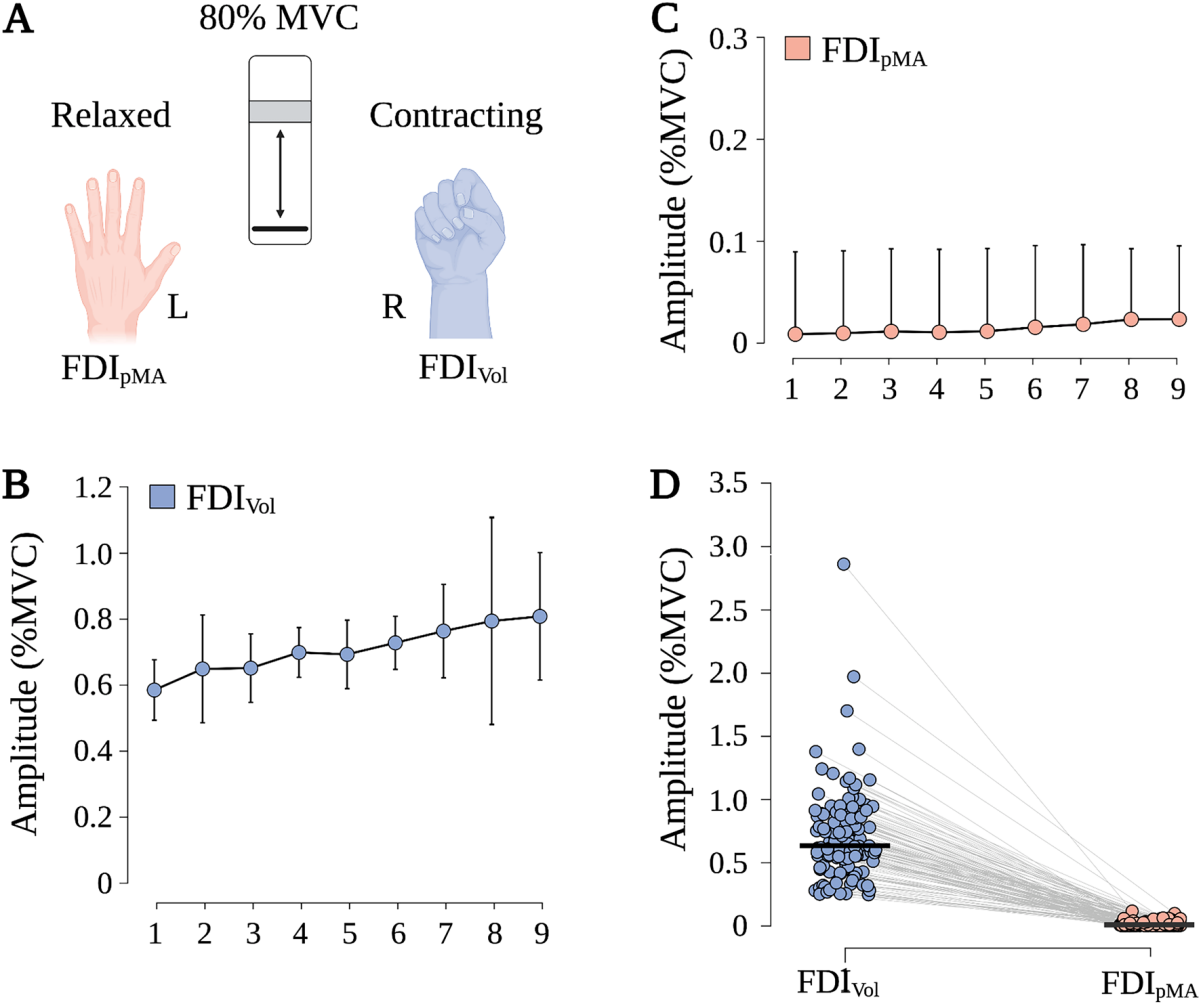
Experimental setup and amplitude of sEMG for FDI_Vol_ and FDI_pMA_. (**A**) Participants performed a unilateral force generation task. Therefore, the actively contracting (right) hand was used to operate a custom-built force sensor to perform an isometric pinch-task for which the thumb and index finger applied force to the force sensor while the resting (left) hand remained relaxed. (**B**) Average MVC-normalized amplitude of FDI_Vol_ of all participants and for all blocks (mean ± 95% confidence interval). (**C**) Average MVC-normalized amplitude of FDI_pMA_ of all participants for all blocks (mean ± 95% confidence interval). (**D**) Raincloud plot of MVC-normalized amplitudes of all 3 s contractions (14 participants × 45 contractions) for FDI_Vol_ and FDI_pMA_. Note the subliminal nature of pMA with amplitudes that are only a fraction of the amplitude of the actively contracting muscle. The black horizontal bar represents the mean. Created with Biorender.com.

Participants performed a unilateral force generation task. Therefore, the actively contracting (right) hand was used to operate a custom-built force sensor to perform an isometric pinch-task for which the thumb and index finger applied force to the force sensor while the resting (left) hand remained relaxed (see Fig. 1A). Force was applied on a single-point load cell (LAUMAS Elettronica Srl, Italy) with a net weight of 0.2 kg and platform dimensions of 25 × 30 × 35 mm. The maximum force capacity of the load cell is 50 kg with a combined error of < ± 0.02%. Participants were instructed to focus solely on the active hand while there was no feedback from ongoing pMA to avoid deliberate inhibition of involuntary muscle activity^33^. The participants were not aware of the study interest at any time during the experiments.

Visual feedback during the pinch force task was provided on a PC using Presentation 16.5 (NeuroBehavioral Systems, Albany, USA). The screen showed a target field and a horizontal bar. The objective was to move the bar as rapidly and accurately as possible into the target field by exerting pressure on the force sensor. The target field, as well as the force required to reach it, were adjusted to individual maximum voluntary force values, representing 80% of maximum force.

A maximum force test was performed separately for both the right and left hands before the test. Participants exerted an individual maximum contraction 3 times (5 s duration for each repetition) with a resting period of 1 min between contractions to counteract fatigue and prevent injuries. The trial where participants exerted the highest force was taken as their individual maximum force. This trial was further used for the normalization of individual force levels during the behavioral task. The corresponding EMG amplitude to this trial was then taken as the individual maximum voluntary contraction (MVC) for further EMG MVC normalization. For this purpose, EMG activity over a window of 500 ms around the peak of the maximum contraction was averaged. The subsequent task used a block design consisting of a constant force level (80% of individual maximum force). Five isometric contractions were performed using the right hand within one block. One contraction lasted for 3 s, with 3 s of rest between contractions. As all participants performed 5 contractions per block, this resulted in a total duration of 30 s per block. Each block was followed by a resting period of 4:30 min to allow partial recovery. In total, each participant completed 9 blocks. Taken together, the time to finish the task was 45 min.

### EMG recordings and analysis

sEMG was recorded on a wireless Desktop Direct Transmission System (NORAXON Inc., Scottsdale, USA). EMG signals were obtained from bilateral first dorsal interossei muscles (FDI) using bipolar surface electrodes (Ag/AgCl; diameter: 10 mm). Inter-electrode distances were consistent at 20 mm and electrodes were attached in parallel to muscle fiber orientation. This configuration allowed us to record sEMG activity over the voluntarily contracting FDI (FDI_Vol_), as well as subliminal pMA over the homologous FDI (FDI_pMA_) of the relaxed hand. EMG data were recorded with a sampling frequency of 1500 Hz, band-pass filtered at 10–500 Hz, input impedance > 100 MΩ, Common Mode Rejection Ratio (CMRR) > 100 dB, and a gain of 500.

All EMG processing was performed using custom-written code implemented in MATLAB (v. R2021b, The MathWorks Inc., Natick, USA). Initially, all EMG data were decimated to 500 Hz. For this purpose, we applied a type I Chebyshev filter (cutoff frequency of 200 Hz) to the data before downsampling to avoid aliasing effects^34^, and subsequently, high-pass filtered EMG signals at 20 Hz (4th order Butterworth filter) to remove low-frequency noise^35^. Additionally, both EMG signals (FDI_Vol_ and FDI_pMA_) were overlaid and time-locked to confirm the preservation of temporal relationships between both signals.

#### Time-domain analysis

Voluntary muscle onsets of FDI_Vol_ were manually defined for each burst through visual inspection of EMG traces performed by a single trained researcher. Participants were instructed to keep all muscles of the upper extremity as relaxed as possible in between every contraction. Onsets of FDI_pMA_ were defined analogously to those of FDI_Vol_. Mean EMG amplitudes of FDI_Vol_, as well as FDI_pMA_, were determined by calculating the root mean square (RMS) values of each burst. To ensure comparable EMG amplitudes, all EMG amplitudes were normalized to individual MVC values (see “Behavioral experiment” section), which were recorded separately for each hand at the beginning of each session. All bursts were subsequently pooled per block (BL 1–BL 9) for each participant and subjected to further statistical analyses.

#### Frequency-domain analysis: spectral features

Initially, power spectral densities (PSD) were estimated according to Welch’s method^36^. To characterize frequency-specific patterns of FDI_Vol_ and FDI_pMA_, four commonly reported frequency features were employed: mean frequency (MNF), median frequency (MDF), peak frequency (PKF), and frequency ratio (FR)^25,37,38^. Both MNF and MDF were estimated using the corresponding MATLAB functions, where MNF is the average frequency obtained by taking the sum of the product of the EMG power spectrum and the frequency and dividing both by the total sum of the power spectrum. MDF is defined as the frequency at which the EMG power spectrum is divided into two regions of equal amplitudes. PKF corresponds to the frequency of maximum energy content of the power spectrum and FR is calculated as a ratio between low- and high-frequency content of the EMG signals. Low- and high cutoff frequencies of FR can be defined either by experiment or MNF^39^. Here, we chose to set low and high frequency cutoffs after careful inspection of power spectral densities to account for the exploratory aspect of FDI_pMA_ frequency content.

#### Frequency-domain analysis: intermuscular coherence

To analyze common input between active and resting hands, IMC was estimated between FDI_Vol_ and FDI_pMA_. Coherence is an extension of Pearson’s correlation coefficient in the frequency domain and describes linear relationships between two input signals. Coherence is obtained through the normalization of the cross-spectra between two signals by their auto-spectra^40^. First, EMG signals were epoched per block and contractions yielding 45 trials (5 contractions × 9 blocks). All EMG bursts were then rectified by way of Hilbert transform. The amplitude of the Hilbert transform provides the envelope of the broadband EMG signal, gives similar results to full-wave rectification, and has been commonly employed to study intermuscular coherence^41,42^. In a final step, bursts were concatenated in a blockwise manner yielding 5 FDI_Vol_ and 5 corresponding FDI_pMA_ bursts per block for each participant. This is done to reduce noise in the signals^22^. After further inspection of EMG signal quality, no bursts were excluded from further analysis, neither for FDI_Vol_ nor for FDI_pMA_. Intermuscular coherence was subsequently estimated between pairs of EMG data (FDI_Vol_ and FDI_pMA_) in this blockwise manner using Welch’s method with a Hanning window of 250 ms without overlap^26^. The significance of IMC results was determined by calculating confidence limits according to Rosenberg et al.^43^:

1 $$CL_{\alpha } = 1 - \left( {1 - \frac{\alpha }{100}} \right)^{{\frac{1}{N - 1}}}$$

where α is the significance set to 5%, N is the number of epochs, and CL reflects the confidence limit above which observed coherence values are deemed significant. IMC estimates were then integrated over four relevant frequency bands: (1) α (8–12 Hz), (2) β (13–30 Hz), (3) γ (30–60 Hz), and (4) γ′ (60–100 Hz) to yield IMC areas and used for further statistical analysis. These frequency bands were selected based on previous studies demonstrating that muscles receive oscillatory neuronal input spanning across these frequencies^44,45^. Analysis of the areas of coherence estimates is considered to be superior compared to analysis of peaks and frequencies of coherence estimates^46–48^.

### Statistical analyses

All statistical analyses were conducted using the free and open-source statistical software program JASP (JASP Team (2021). JASP (Version 0.16.1.0) [Computer software]; Retrieved from https://jasp-stats.org). Since the majority of all variables were normally distributed, as assessed by Shapiro–Wilk testing (α = 0.05), parametric analyses were implemented.

#### Amplitude

Blockwise amplitudes of FDI_Vol_ and FDI_pMA_ were analyzed using a repeated-measures ANOVA with the within-subject factors HAND (FDI_Vol_ and FDI_pMA_), and BLOCK (BL 1–BL 9).

#### Frequency features

Concerning frequency features, blockwise MNF, MDF, PKF, and FR were analyzed using separate repeated-measures ANOVAs with the within-subject factors HAND (FDI_Vol_ and FDI_pMA_), and BLOCK (BL 1–BL 9).

#### Intermuscular coherence

Summed coherence values of each analyzed frequency band (α, β, γ, γ′) were separately assessed using repeated-measures ANOVAs with the within-subject factor BLOCK (BL 1–BL 9).

For all analyses, the statistical threshold was set at p < 0.05. Effect sizes were expressed using partial eta squared (η_p_^2^) for ANOVAs and Cohens d for Bonferroni corrected post-hoc comparisons. A Greenhouse–Geisser correction was implemented when the sphericity assumption of repeated-measures ANOVAs was violated.

## Results

### Amplitude

Repeated measures ANOVA indicated a significant effect for HAND (F_(1, 13)_ = 97.390, p < 0.001, η_p_^2^ = 0.882) on sEMG amplitude (see Fig. 1). Post-hoc comparison showed that sEMG amplitudes of FDI_Vol_ were significantly higher compared to FDI_pMA_ (mean difference (MD) = 69.3%MVC, SE = 7.0%MVC, p < 0.001, d = 2.637). However, no significant effect was found for BLOCK (F_(1.584, 20.598)_ = 1.360, p = 0.274, η_p_^2^ = 0.095) and no significant interaction effect HAND × BLOCK (F_(1.694, 22.016)_ = 1.148, p = 0.327, η_p_^2^ = 0.081) was observed.

### Frequency features

**Figure 2:**
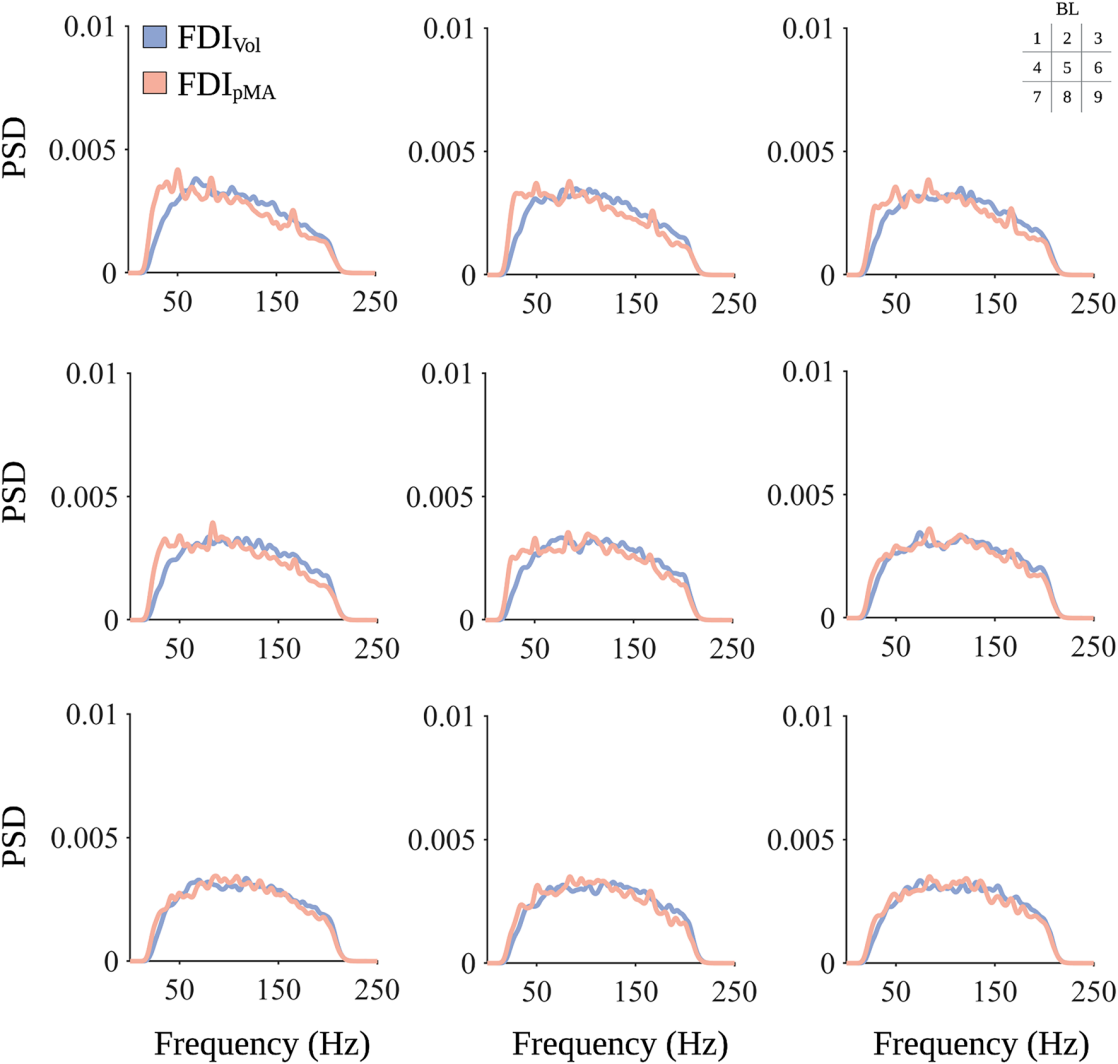
Power spectral density (PSD) for FDI_Vol_ and FDI_pMA_. Displayed are blockwise PSDs averaged across all participants estimated according to Welch’s method. Each subplot represents averaged PSDs for one block (1–9), the order of which is highlighted in the matrix legend in the upper-right corner.

Visual inspection of block-wise PSDs revealed a clear difference between FDI_Vol_ and FDI_pMA_ in the low-frequency range up to 40 Hz (see Fig. 2). Therefore, the cutoff frequency between low and high-frequency components was set at 40 Hz, which was subsequently used to calculate FR. In addition, this cutoff is consistent with previous studies analyzing the relationship of low-frequency and high-frequency signal components in EMG^37^.

**Figure 3:**
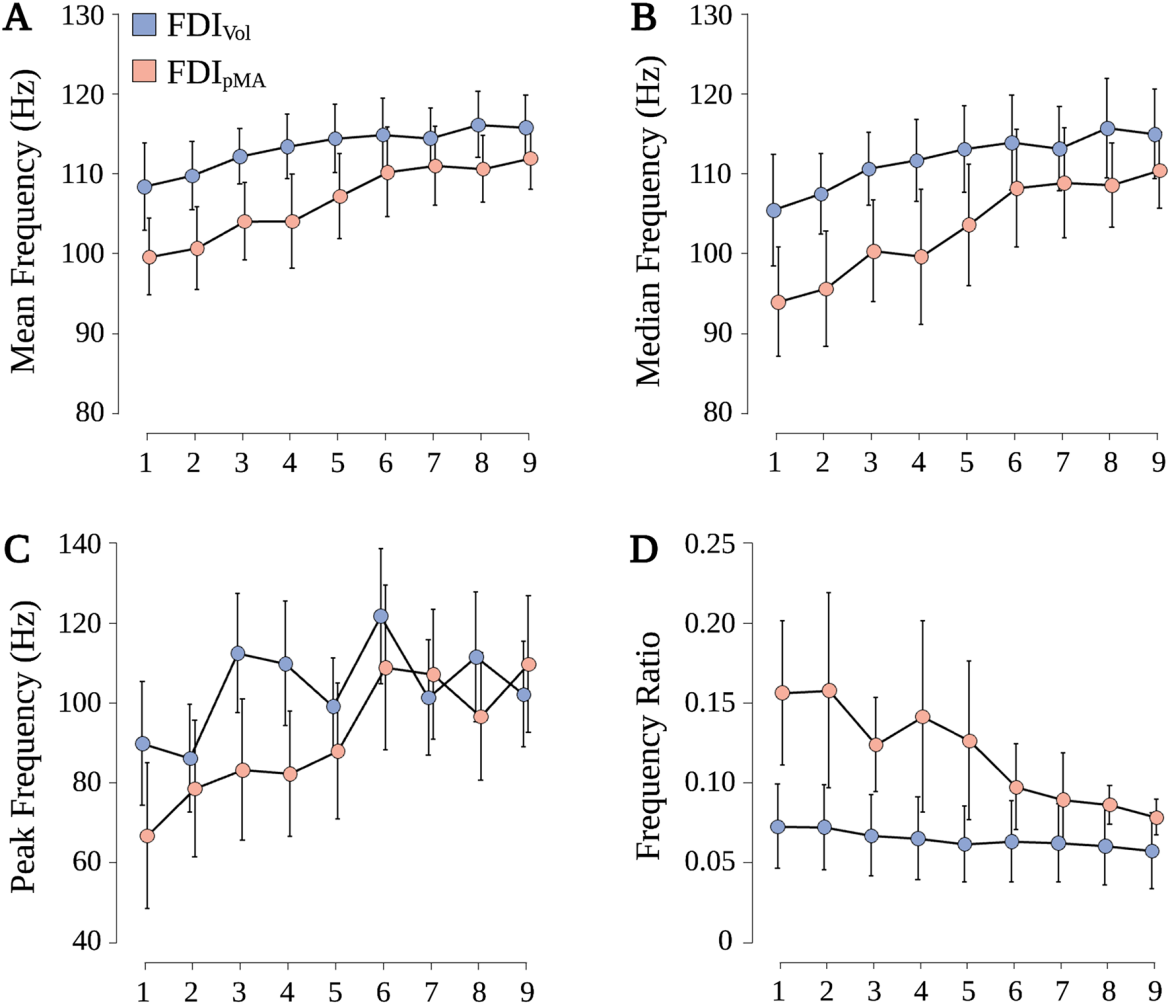
Frequency features of FDI_Vol_ and FDI_pMA_. (**A**) Blockwise mean frequency (MNF). (**B**) Blockwise median frequency (MDF). (**C**) Blockwise peak frequency (PKF) corresponding to the frequency of the maximum energy of the power spectrum. (**D**) Blockwise frequency ratio (FR), calculated as a ratio between low- and high-frequency content of the sEMG signals with a cutoff frequency of 40 Hz. Values are displayed as mean ± 95% confidence interval.

Regarding MNF, repeated-measures ANOVA revealed a significant effect for BLOCK (F_(3.344, 43.467)_ = 15.186, p < 0.001, η_p_^2^ = 0.539; see Fig. 3A). Pairwise post-hoc comparisons indicated that from BL 4 onwards, MNF gradually increased compared to BL 1 (BL 1 vs. BL 4: MD = 4.73 Hz, SE = 1.31 Hz, p = 0.017, d = 0.964). No effect for HAND (F_(1, 13)_ = 3.971, p = 0.068, η_p_^2^ = 0.234) or interaction effect HAND × BLOCK (F_(2.582, 33.564)_ = 1.530, p = 0.228, η_p_^2^ = 0.105) was found.

A similar relation was observed for MDF. Repeated-measures ANOVA showed a significant effect for BLOCK (F_(3.581, 46.657)_ = 13.301, p < 0.001, η_p_^2^ = 0.506; see Fig. 3B). Pairwise post-hoc comparisons revealed that from BL 5 until BL 9, higher MDF compared to BL 1 were observed (BL 1 vs. BL 5: MD = 8.64 Hz, SE = 1.83 Hz, p < 0.001, d = 1.265). No effect for HAND (F_(1, 13)_ = 3.787, p = 0.074, η_p_^2^ = 0.226) or interaction effect HAND × BLOCK (F_(2.763, 35.914)_ = 1.361, p = 0.271, η_p_^2^ = 0.095) was found.

When looking at PKF, repeated-measures ANOVA revealed a significant interaction effect HAND × BLOCK (F_(8, 104)_ = 2.190, p = 0.034, η_p_^2^ = 0.144) and a significant effect for BLOCK (F_(8, 104)_ = 7.043, p < 0.001, η_p_^2^ = 0.351; see Fig. 3C). Simple main effect analysis showed that PKF of FDI_Vol_ was significantly higher than PKF of FDI_pMA_ during BL 1 (89.8 Hz vs. 66.7 Hz, F = 6.596, p = 0.023), BL 3 (112.4 Hz vs. 83.1 Hz, F = 5.863, p = 0.031) and BL 4 (109.8 Hz vs. 82.2 Hz, F = 7.026, p = 0.020). Pairwise post-hoc comparisons for BLOCK indicated that BL 6, BL 7, BL 8 and BL 9 had higher PKF compared to BL 1 (BL 1 vs. BL 6: MD = 37.02 Hz, SE = 6.228 Hz, p < 0.001, d = 1.589; BL 1 vs. BL 7: MD = 25.93 Hz, SE = 6.228 Hz, p = 0.002, d = 1.113; BL 1 vs. BL 8: MD = 25.77 Hz, SE = 6.228 Hz, p = 0.003, d = 1.106; BL 1 vs. BL 9: MD = 27.64 Hz, SE = 6.228 Hz, p < 0.001, d = 1.186). Again, no effect for HAND (F_(1, 13)_ = 2.230, p = 0.159, η_p_^2^ = 0.156) was found.

In terms of FR, repeated-measures ANOVA revealed a significant effect for HAND (F_(1, 13)_ = 5.651, p = 0.033, η_p_^2^ = 0.303) and BLOCK (F_(2.383, 30.980)_ = 5.469, p = 0.007, η_p_^2^ = 0.296; see Fig. 3D). Post-hoc comparison of HAND indicated that FR of FDI_Vol_ was significantly lower compared to FDI_pMA_ (MD = − 0.053, SE = 0.022 Hz, p = 0.033, d = − 0.653). Pairwise post-hoc comparisons of BLOCK revealed that from BL 7 onwards, FR gradually decreased compared to BL 1 (BL 1 vs. BL 7: MD = 0.039 Hz, SE = 0.011 Hz, p = 0.017, d = 0.967). However, no significant interaction effect HAND × BLOCK (F_(2.301, 29.914)_ = 2.850, p = 0.067, η_p_^2^ = 0.180) was found.

### Intermuscular coherence

**Figure 4:**
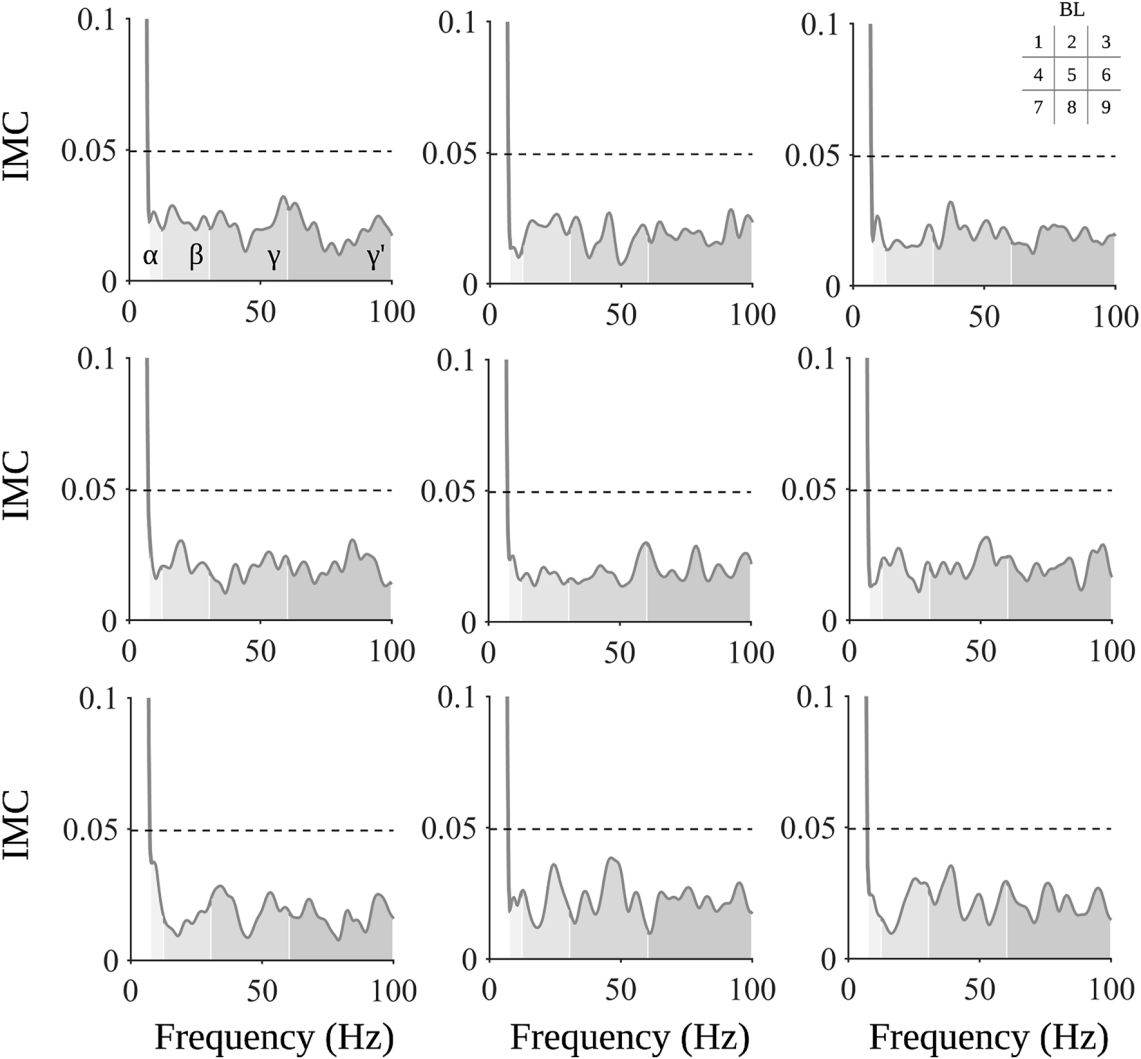
Intermuscular coherence (IMC) between sEMG of FDI_Vol_ and FDI_pMA_. Blockwise averaged spectra of IMC between FDI_Vol_ and FDI_pMA_ suggest the absence of significant coherence for all blocks in broadband sEMG. The confidence limit is indicated by a dashed horizontal line according to Eq. (1). Each subplot represents averaged IMC spectra of all participants for one block (1–9), the order of which is highlighted in the matrix legend in the upper-right corner.

On an individual level, significant IMC was observable in all participants, although with a high inter-subject variability concerning the frequency band and block at which significant IMC was found (please see Fig. S1 for a detailed overview of individual IMC spectra). Critically, pooled IMC spectra between FDI_Vol_ and FDI_pMA_ do not show significant grand-averaged coherence for any block in broadband sEMG (see Fig. 4).

Summed coherence values of the analyzed frequency bands did not show a blockwise modulation as indicated by repeated-measures ANOVAs with non-significant factor BLOCK (α-band: F_(3.198, 41.576)_ = 0.867, p = 0.472, η_p_^2^ = 0.063; β-band: F_(8, 104)_ = 1.070, p = 0.390, η_p_^2^ = 0.076; γ-band: F_(8, 104)_ = 0.727, p = 0.667, η_p_^2^ = 0.053; γ′-band: F_(8, 104)_ = 0.793, p = 0.610, η_p_^2^ = 0.058).

## Discussion

With this study, we aimed to investigate spectral features in active and resting hands during repeated unilateral contractions as well as IMC to potentially reveal common input between active and resting hands. Regarding frequency features of both FDI_Vol_ and FDI_pMA_, we found a significant increase in MNF, MDF, and PKF in both hands from blocks 4, 5, and 6, respectively, compared to block 1. We also observed a significant interaction between the factors HAND and BLOCK for PKF, where PKF was higher in FDI_Vol_ than FDI_pMA_ during block 1 and block 4. No significant differences between both hands were found in either of these measures. For FR, results showed significantly higher values in FDI_pMA_ compared to FDI_Vol_ as well as a gradual decrease in FR of both hands from block 7 to block 9. Intermuscular coherence was evident across participants on an individual level, however, grand-averaged IMC failed to show any significant pattern of common input between both hands. In addition, we integrated IMC across several frequency bands of interest to compare IMC content between blocks of unilateral contractions but again were unable to detect differences in IMC content between different blocks. All findings and their implications are discussed below.

As expected, amplitudes of FDI_Vol_ were significantly higher compared to FDI_pMA_. This is a robust finding as pMA amplitude, if evident, is usually only a fraction of the amplitude of the voluntarily contracting hand^4,49^. One aspect of our approach to improving the understanding of pMA was to study frequency features of pMA. Among the most commonly analyzed frequency features are MNF, MDF, and PKF. In this study, all three features were significantly increased from block 4 (MNF), block 5 (MDF), and block 6 (PKF) onwards compared to block 1 in both FDI_Vol_ and FDI_pMA_. Spectral features are subject to influence by a number of factors. One such factor is fatigue. Many studies report a decrease in MDF and MNF due to muscular fatigue, especially in isometric contractions^25^. This is attributed to the fact that peripheral fatigue leads to a downward shift in the frequency spectrum of sEMG signals due to slower muscle fiber conduction velocity, which results in a compression of the power spectrum and subsequently lower MDF and MNF values^22^. Nevertheless, it is worth noting that some studies show a broad range of MDF during muscular fatigue protocols, which somewhat contextualizes the strength of the association between MDF and muscular fatigue^50,51^. Importantly, the behavioral task employed here was designed to counteract potential fatigue effects by providing numerous rest periods between individual contractions and also between individual contraction blocks. Prolonged rest periods have been shown to be effective in preventing fatigue effects on sEMG signals during repeated contractions^52^. Thus, the increase in MDF and MNF observed in this study suggests that peripheral fatigue did not have a decisive effect on either parameter. Another factor that may modulate MDF and MNF is the force level which can be determined by MVC monitoring. However, the evidence is equivocal. Where some studies reported an increase of both parameters with increasing MVC^50,51^, others observed a decrease^53^ or no modulation between different levels of MVC^54^. Factors such as gender^53^, different muscle fiber compositions^55^, and electrode configurations^25^ have been cited as possible reasons for these varying results. An interesting finding in this context comes from Bilodeau et al.^56^ who were able to show that MDF and MNF increase in some muscles while they decrease in other muscles during contractions with increasing MVC. This implies a muscle-specific modulation of MDF and MNF or at least a considerable variability of these measures between different muscles. Similar findings demonstrate inconsistent patterns of PKF during force level modulations^57^. A further potential factor influencing MDF and MNF is the degree of motor recruitment. Generally, it is assumed that MVC-normalized sEMG signals allow conclusions to be drawn about motor recruitment, although this relationship has not been clearly established. Still, several studies show moderate^58^ to weak^59^ associations between sEMG amplitudes and the recruitment of motor units. The degree of motor unit recruitment may also influence frequency features. For example, a relationship between a linear increase in MDF and orderly recruitment of motor units has been demonstrated in animals^60^. Still, this relationship shows high inter-subject variability in humans^59^. Interestingly, although participants in this study were instructed to contract at 80% of their maximum force, our results show a gradual increase from roughly 60% MVC to 80% MVC from block 1 to block 9 in FDI_Vol_ and a comparable increase, albeit at lower force levels, for FDI_pMA_ (Fig. 1). It is tempting to speculate that the observed increase in MDF and MNF is partly influenced by the increase in muscular activation, i.e., neural drive, as quantified by an increase in MVC in both FDI_Vol_ and FDI_pMA_. Thus, the increase in MDF and MNF observed in FDI_Vol_ and FDI_pMA_ might be explained by progressive recruitment of new motor units with higher muscle fiber conduction velocities as a result of increasing muscle force^60^. Analysis of FR revealed significantly higher FR in FDI_pMA_ compared to FDI_Vol_ as well as a significant decrease in FR from block 7 to block 9. In this study, a higher FR implies a higher proportion of low-frequency (20–40 Hz) signal components. This decrease in FR, similar to the observed changes in MDF, MNF, and PKF, can potentially be associated with increased recruitment of motor units. With increasing recruitment of motor units, the ratio between low- and high-frequency components of the EMG spectrum tilts in favor of the high-frequency components^60,61^. The fact that we observed an increased FR in FDI_pMA_ predominantly in early blocks (Figs. 2, 3D) followed by an approximation of FR between both hands in later blocks may imply an increase in the spillover between FDI_Vol_ and FDI_pMA_ related to the progressive recruitment of motor units in the active hand. Naturally, further evidence is needed to support this assumption. Surface EMG decomposition methods show promising results^59^ and should be considered in future studies investigating pMA mechanisms.

Regarding IMC, we observed two main findings. On an individual level, several participants showed significant IMC spanning from alpha to high gamma frequency bands. This finding is consistent with a previous study evaluating IMC between homologous hand muscles during rhythmic contractions. The authors found coherence between homologous hand muscles across participants although average patterns and their frequency bands were not analyzed^62^. Coherence was in part attributed to so-called neural cross-talk, i.e., patterns of interference between limb movements due to neural interactions between hemispheres, a process mainly mediated through the corpus callosum^63^. Indeed, studies on pMA in hand movements confirm an association between transcallosal fibers connecting bilateral primary motor cortices (M1) and pMA^4^. Specifically, decreased interhemispheric inhibition (IHI) from contralateral to ipsilateral primary motor cortices appears to be associated with increased pMA^64^, suggesting a governing role for the contralateral M1 in the suppression of pMA. However, inhibition appears to decrease with increasing force demands, as the ipsilateral M1 is progressively recruited in the process^65^. Isometric contractions above 30% of MVC have been shown to decrease IHI from contralateral to ipsilateral M1, which in turn allows an increase in corticospinal excitability^66^. In conjunction with our results, it can be speculated that the high contraction force (up to 80% MVC) caused a reduction in the inhibitory influence of the contralateral M1 on the ipsilateral M1, both inducing prominent pMA and serving as an explanation for the common input between the FDI_Vol_ and FDI_pMA_ observed at the participant level. Critically, the patterns of IMC were highly variable between participants and, therefore, did not allow precise conclusions to be drawn about potential neuronal commonalities. Variability was evident both in terms of the blocks and the frequency bands within which IMC occurred. Blockwise grand-averaged IMC did not yield significant results, further emphasizing that precise patterns of common input between FDI_Vol_ and FDI_pMA_ were not detected in this study. In general, these results do not equate to the absence of a common neural origin, but rather imply that the spectral correlates of this common input are highly individual. Future studies should pursue approaches in which pMA is maximized, for example, through fatigue protocols to increase the likelihood of robust patterns of shared input between active and resting muscles. Future studies should extend our results by corticomuscular coherence (CMC) analyses between electroencephalography and EMG of the active and resting hand^67^. CMC is particularly useful for inferring cortical origins in electrophysiological signal pairs and is therefore particularly useful for revealing further evidence regarding the neural origin of physiological mirror activity.

### Limitations

A limitation of the present study is the selection of an all-male participant group. This was done to prevent variance issues due to possible sex differences in central-nervous structure and function^30^ as well as initial findings suggesting sex differences in pMA^68^. For this reason, our findings cannot be applied to female populations in general. Another limitation of our results relates to the age range of the participants. It is known that pMA follows an inverted U distribution as a function of age^68^. For this reason, our results need to be extended to other age ranges. A further influence on pMA results from the sporting activity of the participants. Previous research demonstrated that athletes who perform chronic, organized, high-volume training have higher pMA compared to non-athletes^5^. In this study, we selected participants without organized training backgrounds and low training volumes, corresponding to contemporary health guidelines. It will be interesting to investigate the influence of organized athletic activity at different performance levels on the magnitude as well as neural characteristics of pMA. Furthermore, we investigated only one force level (80% of maximum force) in this study. This was done in light of the positive correlation between pMA amplitude and force level. Nevertheless, future studies should compare different force levels to gain insight into modulatory distinctions of neural input between FDI_Vol_ and FDI_pMA_ relating to force requirements. Ultimately, the present results apply to a single muscle only. Although the FDI is one of the most frequently studied muscles concerning pMA, further studies on neural mechanisms of pMA should also be carried out in other muscles, e.g., proximal muscles of the upper extremity as well as proximal and distal muscles of the lower extremity. This will allow more complete and robust conclusions to be drawn about the characteristics of pMA and its neural origins.

## Conclusion

In summary, our results imply an overlap of spectral properties between active and passive hands during repeated unilateral contractions. This relationship may result from a reduction in interhemispheric inhibition resulting from high force levels as well as progressive recruitment of motor units. However, further studies are needed to support these assumptions. The investigation of pMA holds implications not only for clinical applications but also for understanding bimanual motor control. Our results provide an initial reference point for further investigations. Such studies may extend the inference of muscle activity from global (sEMG) to local (motor unit domain) parameters to understand the underlying processes more precisely. In this sense, the use of EMG decomposition methods is particularly promising.

## Supplementary Information

Supplementary Figure S1.

## Data Availability

Data, in an anonymous format (according to data protection policy in the ethics agreement), is available at 10.6084/m9.figshare.19328747.v1.
