# Supplementary material for: Spectral properties of physiological mirror activity: an investigation of frequency features and common input between homologous muscles
Source: Sci Rep. 2022 Sep 24;12:15965. doi: 10.1038/s41598-022-20413-2 (PMC9509371; doi:10.1038/s41598-022-20413-2)
Supplement: Supplementary file 1 — Supplementary Figure S1. [file 41598_2022_20413_MOESM1_ESM.docx]

Spectral Properties Of Physiological Mirror Activity: An Investigation of Frequency Features and Common Input Between Homologous Muscles

**Rouven Kenville^1,2^***^†^**, Tom Maudrich^1,2^***^†^

**
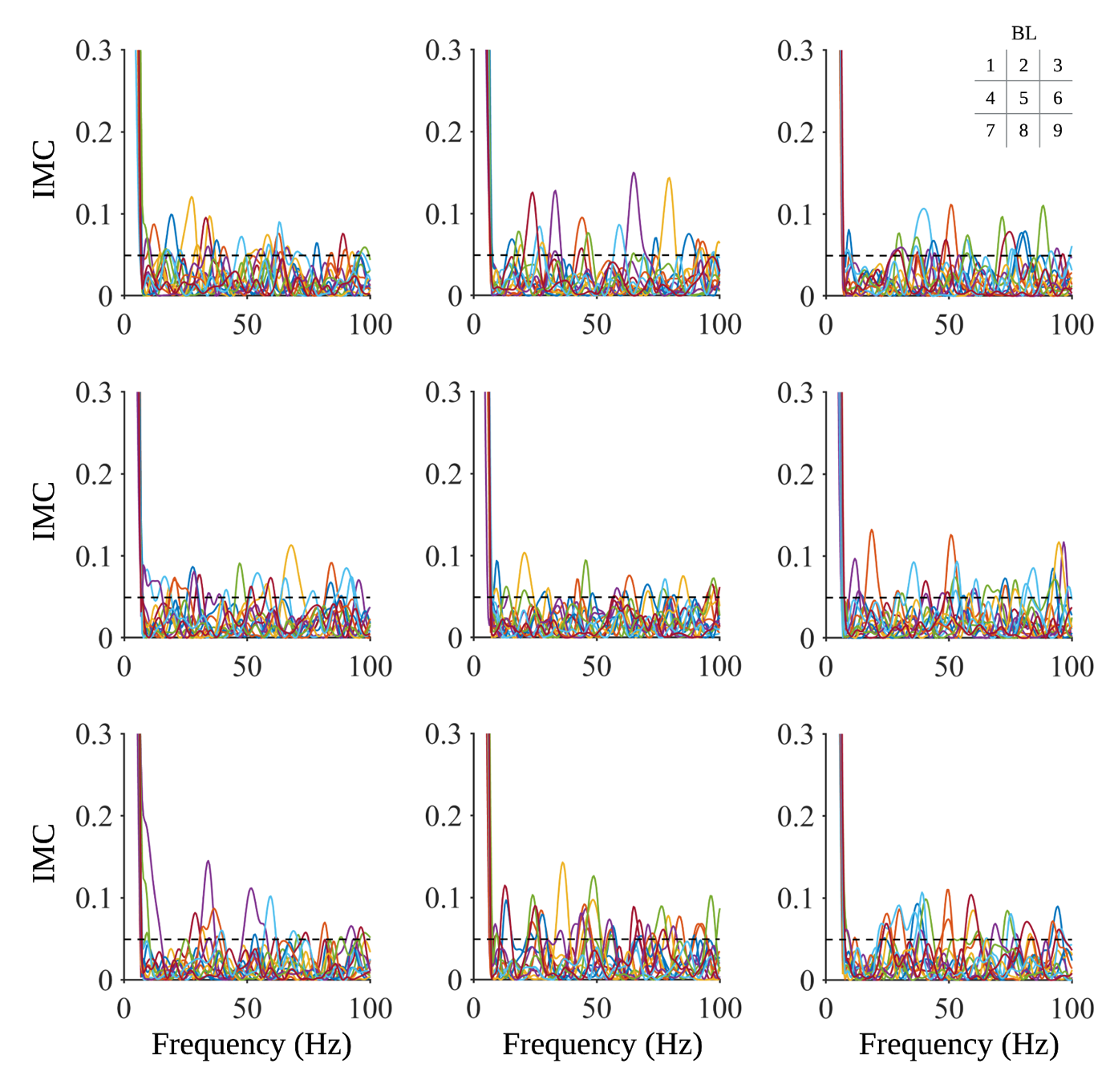
**

**Figure S1. Individual Intermuscular coherence (IMC) between sEMG of FDI_Vol_ and FDI_pMA_.** Blockwise spectra of individual IMC (n = 14) between FDI_Vol_ and FDI_pMA_. The confidence limit is indicated by a dashed horizontal line according to eqn (1).
